# Writing group program reduces academic procrastination: a quasi-experimental study

**DOI:** 10.1186/s40359-021-00665-9

**Published:** 2021-10-12

**Authors:** Bhina Patria, Latifatul Laili

**Affiliations:** 1 Faculty of Psychology, Universitas Gadjah Mada, Bulaksumur, Yogyakarta, 55281 Indonesia; 2 Faculty of Psychology and Socio-Cultural Sciences, Universitas Islam Indonesia, Sleman, Yogyakarta, 55584 Indonesia

**Keywords:** Academic procrastination, Writing group, Thesis

## Abstract

**Background:**

Procrastination is a common problem in higher education. It leads to negative consequences on students’ health and academic achievement. Nevertheless, research concerning interventions has not yet produced consistent results. This study aims to examine the effectiveness of a writing group program on reducing academic procrastination.

**Methods:**

This study was a quasi-experimental study with a one-group pretest-posttest design using double pretests. A double pretest design was used to ensure the internal validity of the experiment. Twenty graduate students followed a 15-days writing group program consisted of a training session and four sessions of writing groups. A thesis procrastination scale was used to measure the intervention’s effects.

**Results:**

The writing group program helped students to set a writing target, discussed writing progress, and provided social support to their colleagues. The results showed that the intervention program could significantly decrease academic procrastination.

**Conclusion:**

The present study demonstrated that a writing group could potentially reduce academic procrastination. Thus, students could benefit from a writing group when working on their master thesis. A thesis preparation course that provides information about goal-setting strategy and the principles of effective writing habits (i.e., behavioral, artisanal, social and emotional habits), might also assist students in writing their thesis. Further research is needed, preferably through the provision of a control group, a randomized assignment and a larger sample.

## Background

Academic procrastination is common among students in higher education. Studies showed that about half of undergraduates and graduate students were procrastinating on academic tasks [1, 2]. Steel and Klingsieck [3] defined academic procrastination as intentionally delaying completion of an assignment related to learning-behavior despite its negative consequences. Final year students show higher levels of procrastination than first-year students [4, 5]. Around 40–60% of graduate students are procrastinated on writing a term paper, studying for examinations, and weekly reading assignments [1]. A cross sectional study revealed that graduate students procrastinated more on writing term papers than other tasks [6]. Solomon and Rothblum [4] also found approximately 45% students procrastinated on completing their term papers. Another study revealed that students in second year and third year are more procrastinated on completing the task than first year student [7]. Puspitasari [8] reported that the majority of graduate students mostly procrastinate on their master thesis. Most of them (94%) have a high or average level of procrastination.

A Master Thesis is a complex and long term assignment. Students develop scientific ideas and research methods using statistical analysis into a manuscript using a specific academic style [9]. Graduate students are more prone to procrastination, especially at the end of their study while writing their thesis. Unlike short term project (e.g. writing a term paper, quiz, reading report), in a long term project, people optimize their effort at the beginning, but along the way, they tend to either not finish or delay their tasks [10]. According to behavioral approach, procrastinator prefer to choose short term rewards so they get immediate pleasure than have long term task [11].

Studies showed that procrastination has negative consequences on students’ health and academic achievement. Procrastination is associated with poor academic performance [12], academic misconduct [13], burnout [14], and depression [15, 16]. Apart from these negative consequences, studies designed to study how to decrease procrastination are still scarce [3, 17, 18]. This study, therefore, aimed to develop an intervention for academic procrastination.

Thesis writing is not only a complicated process but also an individual assignment [19]; therefore, it needs sufficient social support. A study found that loneliness could increase students’ procrastination [20]. Dupont et al. [21] stated that there is a frustration period when completing a thesis that makes students feel lonely and might lead them to give up their studies. Furthermore, when writing a thesis, students reported that they felt anxious and disappointed with their writing [22].

Results from earlier studies demonstrated a strong and consistent association between social support, goal setting, and lower levels of procrastination. Setting proximal goals could reduce procrastination [23]. Another study also found that goal clarification decreased impulsiveness and academic procrastination [24]. Social support from peers significantly decreases procrastination [21, 25]. One way to reduce procrastination is to use social support systems by developing a writing group.

A writing group is an environment that provides opportunities to share writing needs and concerns and to get constructive feedback and support [26–28]. Students can tell their writing goals, obstacles while pursuing the goals, listen and give support to others. A writing group produces several positive outcomes. It improves students’ writing proficiency [26, 29] and thus improves students’ confidence in writing [30]. A writing group also improves motivation, attachment, and cooperation [31, 32]. However, previous studies have not examined the effectiveness of a writing group on reducing procrastination. The purpose of this study is to investigate the effectiveness of a writing group program on reducing students’ academic procrastination. Given that writing group produces several positive outcomes—such as the provision of feedback and support; improves students’ writing proficiency, confidence, and motivation—it is expected that the intervention program will decrease students’ academic procrastination.

## Method

### Participant

This quasi-experimental study involved 20 graduate students of psychology—sixteen female and four male. The students were working on their thesis in the third (*n* = 10), fourth (*n* = 8), or fifth (*n* = 2) semester. The average age was 27.5 (*SD* = 5.16) years old.

### Procedure

This study evaluated a one-group of 20 graduate students in a pretest-posttest study design. Participants followed a writing group program, namely GROWTH or Group for Writing Thesis. A double pretest design was used to ensure there was no threats to internal validity, i.e., maturation, testing, and regression artifacts [33]. A double pretest design is an improved version of one-group pretest-posttest design [33, p. 110]. The double pretest design ensures a better causal inference because the condition between Pretest 1 and Pretest 2 could be regarded as a control condition. While the condition between Pretest 2 and Posttest 1 could be regarded as an intervention condition. Causal relationship could be inferred when there is no significant difference between Pretest 1 and Pretest 2, and there is a significant difference between Pretest 2 and Posttest 1. A second posttest was added to investigate the delayed effect of the intervention.

The intervention was developed based on motivation, goal-setting, and group-support theories [9, 34, 35]. The program consisted of five sessions, which lasted for 15 days. Before beginning the experiment, ethical clearance was sought from the faculty’s institutional review board.

The GROWTH program consisted of one training session followed by four sessions of writing groups. The writing groups session were conducted twice a week. Each group session consisted of three parts: a preface, a group learning process, and a concluding session. Participant were divided into five writing groups based on the similarity of the thesis research method, i.e., qualitative and mixed method (2 groups), experimental study (1 group), correlational study (1 group), and scale development (1 group). The program was led by a facilitator and assisted by five co-facilitators (one for each writing group). There was also an observer for each writing group. The observers monitored the process of the intervention, participant’s reaction, and evaluate whether the goals of the writing group session was achieved.

A training for the facilitator, co-facilitators, and observers were conducted to make sure the process of intervention in each group was standardized. In the training, they learned about the intervention procedure. A try-out was also held where the facilitator and co-facilitators performed the intervention and evaluated the protocol. The intervention timeline are provided in Table 1. The total duration of the intervention meeting was 16 h 30 min. This consisted of 5 h 45 min of the training session and four group meetings; each lasted for 135 min. The total cost for the intervention was $1,300; this includes the $350 cost for refreshments. The interval between Pretest 1 and Pretest 2 was 4 days. Pretest 2 was administered in the first day of the intervention. Posttest 1 was administered right after the completion of the intervention—14 days after Pretest 2.

**Table 1:** Intervention timeline

Time	Session	Activities
Day 1	Training session	Participants set SMART (Specific, Measurable, Achievable, Realistic, and Time based) goals. Participants assessed their BASE (Behavior, Artisanal, Social, and Emotional) Habits. Participants developed writing habits and routines. Participants understood the procedure and rules of writing group session.
Day 2–4; 5–7; 9–11; and 12–14	Self reflection (individual)	Participants worked independently on their thesis, wrote a daily reflective journal, wrote a specific target on a particular day, report achievement of the target, and described the writing process
Day 4, 8, 11, and 15	Group session	Participants shared their writing experience and challenges and whether their targets were achieved or not. Participants gave feedback and support each other

In the training session, the students were trained to set SMART (Specific, Measurable, Achievable, Realistic, and Time based) goals, to assess their BASE (Behavior, Artisanal, Social, and Emotional) Habits [34, 35], and to develop writing habits and routines. The students also learned how a writing group worked.

After the training session, the students were expected to work independently on their thesis, write a daily reflective journal to monitor themselves, write a specific target on their thesis that they want to achieve on a particular day, report whether the target was met or not, and how the writing process went. The writing group sessions consisted of three parts: a preliminary session, a group session, and a concluding session. The facilitator led the preface and conclusion session in a large group, while the individual group sessions were led by co-facilitator in small groups. In the small group sessions, the students shared their writing experience and challenges and whether their targets were achieved or not. The co-facilitator encouraged the students to give feedback and to support each other during the session. At the end of the session, the co-facilitator highlighted the students’ progress. This was important to make the students focus on their growth, feel safe, and comfortable in the group.

### Data collection and instruments

The procrastination scale was modified from the measurement used in previous studies [8, 36]. The scale was self reported consisting four aspects of procrastination—behavior, cognitive, affective, and motivational. There were 25 items with five options, ranging from ‘very unlikely’ to ‘very likely’. This modified version is valid and reliable based on the Aiken’s V coefficient (0.56–0.94) and Cronbach’s Alpha coefficient (0.845).

### Data analysis

To assess the effectiveness of the intervention, we compared the results of the procrastination scale before and after the training. The data were analyzed using ANOVA (Analysis of Variance) repeated measures [37]. The hypothesis would be answered by comparing the second pretest (Pretest 2) scores with posttest scores. The scores should decrease after the intervention so that it could be concluded that the hypothesis was accepted. The comparison between Pretest 1 and Pretest 2 was to assure the internal validity [33]. The comparison between Posttest 1 and Posttest 2 scores was also analyzed to investigate the delayed effects of the intervention after two weeks.

## Results

Table 2 shows that the highest level of procrastination is found in Pretest 2, while the lowest is in Posttest 2.

**Table 2:** The level of procrastination in each observation

Procrastination	Mean	SD
Pretest 1	69.30	9.47
Pretest 2	72.30	11.21
Posttest 1	67.30	11.27
Posttest 2	62.00	9.21

**Fig. 1:**
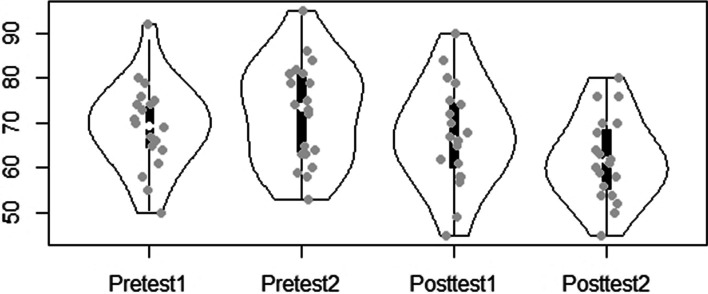
Violin plot of academic procrastination. Violin plot of academic procrastination

**Fig. 2:**
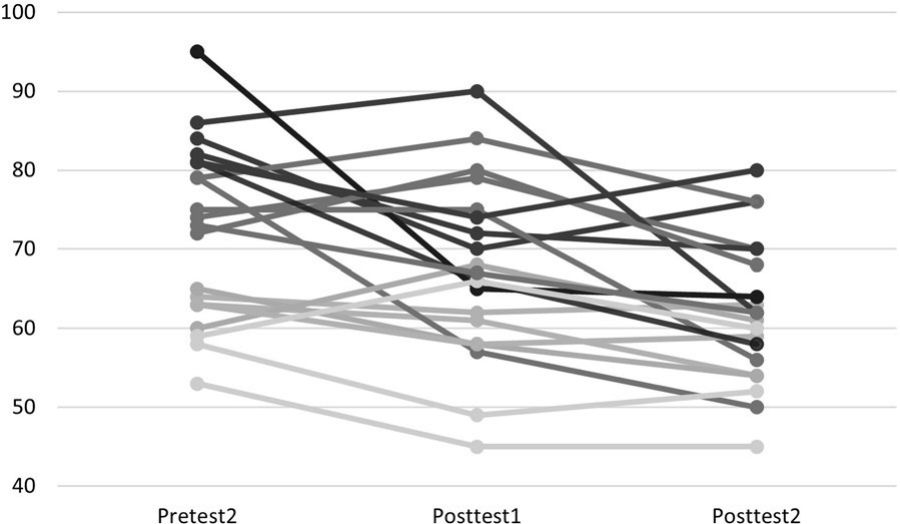
Level of academic procrastination. Level of academic procrastination with individual data points

Figures 1 and 2 depicts the procrastination scores before and after the intervention. There is a slight increase in the procrastination levels from Pretest 1 to Pretest 2. The figure also indicates a decrease in procrastination level after the intervention—between Pretest 2 and Posttest 1. The decrease continues from Posttest 1 to Posttest 2 at a nearly similar point difference.

To assess the differences between the mean scores (Pretest 1, Pretest 2, Posttest 1, and Posttest 2), repeated-measures ANOVA was used. The post-hoc comparisons was calculated with Bonferroni method. Maulchly’s test was conducted to check whether the data violates the assumption of sphericity. The result showed that the assumption of sphericity was violated, $X^2(5) = 12.169$, *p* = 0.033. To produce a valid *F*-ratio, a correction based on the Greenhouse-Geisser estimate of sphericity was conducted $(\epsilon = 0.688 )$ [37, 38]. The results show that there was a significant effect of writing group program on procrastination *F*(2.065, 39.226) = 7.854, p = 0.001.

The results of post hoc tests (Table 3) showed a significant difference in academic procrastination levels between Pretest 2 and Posttest 1 (*MD* = $-5$, *p* = 0.004), between Posttest 1 and Posttest 2 (*MD* = $-5,3,$ p = 0.01), and between Pretest 2 and Posttest 2 (*MD* = $-10.30$, *p* = 0.001). This indicated that the intervention program significantly reduced academic procrastination. Additionally, there was no significant difference between Pretest 1 and Pretest 2 (*MD* = 3.00, *p* = 0.049), which indicated there were no threats to internal validity, i.e., maturation and regression artifact.

**Table 3:** Pair comparison of procrastination level

Comparisons	*p*	Mean difference*	95% Confidence interval			
			Std. error	Lower bound	Upper bound	Effect size
Pretest 1 versus Pretest 2	0.05	3.00	1.429	− 7.207	1.207	
Pretest 2 versus Posttest 1	0.04	− 5.00	2.271	− 1.686	11.686	0.511
Posttest 1 versus Posttest 2	0.01	− 5.30	1.812	− 0.035	10.635	0.542
Pretest 2 versus Posttest 2	0.001	− 10.30	2.199	3.825	16.775	1.053

*Bonferroni corrected

The effect size of the contrast—Pretest 2 versus Posttest 2—was calculated based on the mean squares of the intervention program and the mean squares of the error term [37, 39]. The result showed that $r_{contrast} = 1.053$; this represented a large effect [37]. Therefore the effects of the intervention program on academic procrastination represented a significant finding.

## Discussion

This quasi-experiment confirmed that writing group intervention program—Group for Writing Thesis or GROWTH—could potentially reduce academic procrastination. This result may be explained by the intervention program that was focused on three main activities that reduce academic procrastination, i.e., training, participation in a writing group, and independent activities. In the training session, the students learned the nature of a thesis, SMART (Specific, Measurable, Attainable, Realistic, Time-based) goals, and BASE Habits. These activities made the students aware that writing a master thesis is a long process. It took a lot of time and energy, so students had to apply specific strategies. The strategy used in the training was to breaking down thesis writing into smaller task lists. These lists became short term targets to guide students as they wrote. According to Mühlberger and Traut-Mattausch [40], target development is effective for diminishing procrastination. The target forming helps to bridge the gap between intention and task accomplishment [41]. Svartdal et al. [42] said that when people have more available time to do the task, procrastinators tend to delay the task. In this training, the target enacts as a barrier or a reminder for not delaying the task.

SMART goals, furthermore, were applied to make thesis writing more structured. Students set short term targets, specified the necessary activities and their duration, prioritized the activities, and finally organized all of them into a daily schedule. Students shared and gave feedback to one another in their groups. They had to make sure that the target and the schedule were clear and achievable. A previous study showed that SMART goals could decrease procrastination effectively [24].

In the training session, students also learned about new writing habits. The BASE habits—consisted of behavioral, artisanal, social, and emotional habits—are the main pillars of writing productivity [34, 35]. This concept facilitated students to evaluate their strengths and to use it to overcome academic procrastination. Previous studies showed that a personal strengths-based approach was effective in overcoming academic procrastination [43, 44]. Awareness toward personal strengths was crucial in evaluating students’ current state of mind. They could optimize their strengths by maintaining activities that utilized their strengths in their daily writing routine.

On the other hand, students could evaluate their weaknesses based on BASE habits and act upon them. For example, some students realized that they lacked in social habits and concentration while working on their thesis. The students afterward increased activities related to emotional habits, e.g., practicing relaxation or sharing their difficulties when working on their thesis. Meanwhile, some students found themselves good at artisanal habits. They could maintain this by joining a research workshop, discussing with an experienced researcher, and reading books about scientific writing.

Groups for Thesis Writing inspired students to benefit from the social environment by having regular meetings. Instead of viewing thesis writing as an individual activity, students could get mental and academic support from other group members. A growing body of research showed the positive impacts of social support on procrastination [21, 45, 46]. Nichols and Jenkinson [47] mentioned four characteristics of an effective support group, such as (1) the decrease of isolation through social interaction, (2) chance to release emotion and discussion, (3) conversation leading to more constructive and balanced perspective, and (4) improvement of coping skills through learning from member experiences. Group for Writing Thesis intervention fulfilled those four characteristics.

After setting goals and daily activities, the next step was implementing it into action. This action was the most critical process. As individuals, students tried to stick with their goals and daily planning. Sometimes they succeeded in reaching their target, but sometimes they found it hard to achieve it. While the students were writing a thesis, they faced some unplanned tasks, distractions, and obstacles along the way. König et al. [48] mentioned that setting goals are not enough to change behavior; goal adaptation is essential. Goal adaptation emphasizes the value of flexibility; it gives someone a chance to reevaluate the target, whether it is still relevant to him or her, or needs to be modified. This adaptation is also an opportunity to get creative, seeking another way to solve the difficulties.

The students monitored their actions every day. They wrote their daily targets and decided when they should achieve it. They also reflected on their experience by writing their feelings, thinking, challenges, and opportunities. Some students wrote about their feeling of failure when the target was unmet. When people could not meet the goals, it might lead them to have negative feelings such as powerlessness and the fear of failure.

Walker [46] highlighted that procrastination treatment should be focused on the powerlessness experience. In this study, students expressed that in the past, they had set goals and monthly plans, but often failed to implement them. They thought that setting goals was fruitless, gave up on planning, and procrastinated. According to Haghbin et al. [49], the fear of failure could contribute to procrastination. In this writing group, students evaluated their goal achievement, reflected on the obstacles before them, and set new targets. This process encouraged them to achieve their goals, and they could modify or start a new target instead of giving up. The students also received positive feedback and support from other members of the group. This positive environment helped the students view failure as part of the nature of writing a thesis.

On the other hand, daily monitoring encouraged the students to focus more on the process and less on the outcome; therefore, they experienced daily success instead of feeling a failure when the goals were not achievable. Krause and Freund [50] believed that being process-focused was more helpful in reducing the fear of failure and keeping students in pursuit of their goals rather than being outcome-focused.

The participants in this study were graduates students from three different cohorts. Therefore, there could be an effect of the cohort on academic procrastination. Even though they were from different cohorts, they shared a similar characteristic—they were working on their master thesis. Further analysis also showed that there is no difference in academic procrastination between those cohorts. Consequently, we have no reason to believe that the participants’ cohort matters. Due to the limited sample size, caution must be applied, as the findings might not apply to broader population. Generalization should be limited to the participant, treatment, output, and setting that similar to this study [50]. To conclude, the present study demonstrated that a writing group could potentially reduce academic procrastination. Thus, students could benefit from a writing group when working on their master thesis. A thesis preparation course that provides information about BASE habits and SMART goals could also assist students in writing the thesis. There is abundant room for further progress in determining the effects of each components of the intervention program on procrastination level. For example, further research should be undertaken to investigate whether writing a daily reflective journal had a dominant effects on procrastination level compared to other components. Additionally, future investigations aiming for a better causal inference would benefit from having a control group, a randomized assignment, and a larger sample.

## Data Availability

The data sets used and/or analysed during the current study are available from the corresponding author on reasonable request.

## Abbreviations

ANOVA: Analysis of variance
BASE Habits: Behavior, artisanal, social, and emotional
GROWTH: Group for writing thesis
SMART: Specific, measurable, attainable, realistic, time-based

## References

[1] Onwuegbuzie AJ (2004). Academic procrastination and statistics anxiety. Assess Eval High Educ.

[2] Ozer BU, Demir A, Ferrari JR (2009). Exploring academic procrastination among Turkish students: possible gender differences in prevalence and reasons. J Soc Psychol.

[3] Steel P, Klingsieck KB (2016). Academic procrastination: psychological antecedents revisited. Aust Psychol.

[4] Solomon L, Rothblum E (1984). Academic procrastination: frequency and cognitive-behavioral correlates. J Couns Psychol.

[5] Stewart M, Stott T, Nuttall A-M (2016). Study goals and procrastination tendencies at different stages of the undergraduate degree. Stud High Educ.

[6] Özer B (2011). A cross sectional study on procrastination: who procrastinate more?.

[7] Stewart M, Stott T, Nuttall A-M (2016). Study goals and procrastination tendencies at different stages of the undergraduate degree. Stud High Educ.

[8] Puspitasari A. Peran kecemasan akademik sebagai mediator hubungan antara perfectionism dan prokrastinasi mengerjakan tesis. Thesis, Universitas Gadjah Mada, 2018

[9] Silvia PJ (2007). How to write a lot: a practical guide to productive academic writing.

[10] O’Donoghue T, Rabin M. Procrastination on long-term projects. J Econ Behav Org. 2008;66:161–75. 10.1016/j.jebo.2006.05.005.

[11] Siaputra IB (2010). Temporal motivation theory: best theory (yet) to explain Procrastination. ANIMA Indones Psychol J.

[12] Kim KR, Seo EH (2015). The relationship between procrastination and academic performance: a meta-analysis. Personal Individ Differ.

[13] Patrzek J, Sattler S, van Veen F, Grunschel C, Fries S (2014). Investigating the effect of academic procrastination on the frequency and variety of academic misconduct: a panel study. Stud High Educ.

[14] Balkis M. The relationship between academic procrastination and students’ burnout. Hacet Üniv Eğitim Fak Derg [Hacettepe Univ J Educ]. 2013;28:68–78.

[15] Flett AL, Haghbin M, Pychyl TA (2016). Procrastination and depression from a cognitive perspective: an exploration of the associations among procrastinatory automatic thoughts, rumination, and mindfulness. J Ration Emot Cognit Behav Ther.

[16] Constantin K, English MM, Mazmanian D (2018). anxiety, depression, and procrastination among students: rumination plays a larger mediating role than worry. J Ration Emot Cognit Behav Ther.

[17] Glick DM, Orsillo SM (2015). An investigation of the efficacy of acceptance-based behavioral therapy for academic procrastination. J Exp Psychol Gen.

[18] Rozental A, Forsell E, Svensson A, Andersson G, Carlbring P (2015). Internet-based cognitive-behavior therapy for procrastination: a randomized controlled trial. J Consult Clin Psychol.

[19] Single PB (2010). Demystifying dissertation writing: a streamlined process from choice of topic to final text.

[20] Andangsari EW, Djunaidi A, Fitriana E, Harding D (2018). Loneliness and problematic internet use (piu) as causes of academic procrastination. Int J Soc Sci Stud.

[21] Dupont S, Meert G, Galand B, Nils F (2013). Postponement in the completion of the final dissertation: an underexplored dimension of achievement in higher education. Eur J Psychol Educ.

[22] Fritzsche BA, Young BR, Hickson KC (2003). Individual differences in academic procrastination tendency and writing success. Person Individ Differ.

[23] Wolters CA (2003). Understanding procrastination from a self-regulated learning perspective. J Educ Psychol.

[24] Muñoz-Olano JF, Hurtado-Parrado C (2017). Effects of goal clarification on impulsivity and academic procrastination of college students. Rev Latinoam Psicol.

[25] De Clercq M, Devos C, Azzi A, Frenay M, Klein O, Galand B (2019). I need somebody to lean on. Swiss J Psychol.

[26] Li LY, Vandermensbrugghe J (2011). Supporting the thesis writing process of international research students through an ongoing writing group. Innov Educ Teach Int.

[27] Qunayeer HSA (2020). Supporting postgraduates in research proposals through peer feedback in a Malaysian university. J Furth High Educ.

[28] Mochizuki N. The lived experience of thesis writers in group writing conferences: the quest for “perfect” and “critical.” J Second Lang Writ. 2019;43:36–45. 10.1016/j.jslw.2018.02.001.

[29] Aitchison C (2009). Writing groups for doctoral education. Stud High Educ.

[30] Larcombe W, McCosker A, O’Loughlin K. Supporting education PHD and DED students to become confident academic writers: an evaluation of thesis writers’ circles. J Univ Teach Learn Pract. 2012;4.

[31] Denman PM, Corrales JM, Smyth S, Craven K (2018). From ABD to PHD: a qualitative study examining the benefits of a support group during dissertation in an online doctoral program. J Contin High Educ.

[32] Hass S. A writer development group for master’s students: procedures and benefits. J Acad Writ. 2011;1(1):88–99. 10.18552/joaw.v1i1.25.

[33] Shadish WR, Cook TD, Campbell DT. Experimental and quasi-experimental designs for generalized causal inference, pp. 623623. Houghton, Mifflin and Company, Boston, MA, US; 2002.

[34] Sword H, Sorrenson P, Ballard M (2019). Base pleasures: the behavioural, artisanal, social and emotional dimensions of academic writing. Stud High Educ.

[35] Sword H (2017). Air & light & time & space: how successful academics write.

[36] Sokolowska J. Behavioral, cognitive, affective, and motivational dimensions of academic procrastination among community college students: a q methodology approach. Thesis, Fordham University (2009). https://fordham.bepress.com/dissertations/AAI3361366.

[37] Field A (2013). Discovering statistics using IBM SPSS statistics.

[38] Field A, Hole G (2003). How to design and report experiments.

[39] Salkind NJ (2010). Measures of effect size.

[40] Mühlberger MD, Traut-Mattausch E (2015). Leading to effectiveness: comparing dyadic coaching and group coaching. J Appl Behav Sci.

[41] Gustavson D, Miyake A (2017). Academic procrastination and goal accomplishment: a combined experimental and individual differences investigation. Learn Individ Differ.

[42] Svartdal F, Granmo S, Færevaag F (2018). On the behavioral side of procrastination: exploring behavioral delay in real-life settings. Front Psychol.

[43] Schouwenburg HC. In: Schouwenburg, H.C., Lay, C.H., Pychyl, T.A., Ferrari, J.R. (eds.) On counseling the procrastinator in academic settings. American Psychological Association, Washington, DC (2004). 10.1037/10808-000. http://content.apa.org/books/2004-14505-000.

[44] Visser L, Schoonenboom J, Korthagen FAJ (2017). A field experimental design of a strengths-based training to overcome academic procrastination: short- and long-term effect. Front Psychol.

[45] Eggens L, van der Werf MPC, Bosker RJ (2008). The influence of personal networks and social support on study attainment of students in university education. High Educ.

[46] Walker LJS. In: Schouwenburg, H.C., Lay, C.H., Pychyl, T.A., Ferrari, J.R. (eds.) Overcoming the patterns of powerlessness that lead to procrastination, pp. 75–89. American Psychological Association, Washington, DC; 2004.

[47] Nichols K, Jenkinson J (2006). Leading a support group: a practical guide.

[48] König C, Eerde W, Burch A (2010). Predictors and consequences of daily goal adaptation: a diary study. J Pers Psychol.

[49] Haghbin M, McCaffrey A, Pychyl TA (2012). The complexity of the relation between fear of failure and procrastination. J Ration Emot Cognit Behav Ther.

[50] Krause K, Freund AM (2014). How to beat procrastination: the role of goal focus. Eur Psychol.

